# Effects of GLP-1 Analogues and Agonists on the Gut Microbiota: A Systematic Review

**DOI:** 10.3390/nu17081303

**Published:** 2025-04-09

**Authors:** Krzysztof Ksawery Gofron, Andrzej Wasilewski, Sylwia Małgorzewicz

**Affiliations:** 1 Student Scientific Circle at Department of Clinical Nutrition, Medical University of Gdańsk, Marii Skłodowskiej-Curie 3a, 80-210 Gdańsk, Poland; 2 Student Scientific Association of Medical Chemistry and Immunochemistry, Wroclaw Medical University, Marii Skłodowskiej-Curie 48/50, 59-369 Wroclaw, Poland; andrzej.wasilewski@student.umw.edu.pl; 3 Department of Clinical Nutrition, Medical University of Gdańsk, Marii Skłodowskiej-Curie 3a, 80-210 Gdańsk, Poland; sylwia.malgorzewicz@gumed.edu.pl; 4 Department of Nephrology, Transplantology, and Internal Medicine, Medical University of Gdańsk, Marii Skłodowskiej-Curie 3a, 80-210 Gdańsk, Poland

**Keywords:** GLP-1 analogues, GLP-1 agonists, microbiome, microbiota, obesity

## Abstract

Background: GLP-1 analogues are a relatively new class of medications that form the cornerstone of diabetes treatment. They possess invaluable glucose-lowering properties without hypoglycemic effects as well as strong cardioprotective effects. The gut microbiome has become the focus of numerous studies, demonstrating its influence not only on the gut but also on the overall well-being of the entire body. However, the effects of GLP-1 analogs on gut microbiota remain uncertain. Scope of review: Our systematic review (based on PRISMA guidelines) aimed to gather knowledge on the effects of GLP-1 analogue medications on the composition, richness, and abundance of gut microbiota in both animal and human models. Conclusions: Thirty-eight studies were included in this systematic review. GLP-1 analogues have demonstrated a notable impact on the composition, richness, and diversity of gut microbiota. We can conclude, following the obtained research results of our study, that liraglutide promotes the growth of beneficial genera relevant for beneficial metabolic functions. Exenatide and exendin-4 administration showed various effects on the microbiome composition in animal and human studies. In animal models, it increased genera associated with improved metabolism; however, in human models, genera linked to better metabolic functions and escalated inflammation increased. Following dulaglutide administration, increases in *Bacteroides*, *Akkermansia*, and *Ruminococcus*, genera connected to an improved metabolic model, were significant. Finally, varied results were obtained after semaglutide treatment, in which *A. muciniphila*, known for its positive metabolic functions, increased; however, microbial diversity decreased. Semaglutide treatment provided various results indicating many confounding factors in semaglutide’s impact on the gut microbiota. Results varied due to dissimilarities in the studied populations and the duration of the studies. Further research is essential to confirm these findings and to better recognize their implications for the clinical outcomes of patients.

## 1. Introduction

GLP-1 analogues and GLP-1 receptor agonists are relatively new drugs that are now gaining popularity. In addition to their main effect of treating type 2 diabetes mellitus (T2D), they are recommended for treating chronic kidney disease (CKD) co-morbid to T2D and lowering the risk of cardiovascular events in at-risk groups [1]. Guidelines from the American Diabetes Association recommend the use of GLP-1RA regardless of metformin intake and in patients who need to reduce weight—especially those prone to hypoglycemia [2]. A particularly important effect and recommendation is the use of these drugs in patients at risk of hypoglycemia, as this condition is a significant and potentially fatal consequence of treating diabetes with drugs such as insulins or sulfonylurea derivatives, which are now going out of use [3]. It is important to emphasize the fact that the indications do not include only T2D but are registered and play an important role in the treatment of obesity as an independent disease entity [4,5]. Thus, demonstrating a preventive effect against the potential consequences, complications, and properties of obesity disease and metabolic syndrome [6]. Given the totality of the cited gains and indications, GLP-1 analogues and their receptor agonists represent an important role in the modern treatment of T2D and obesity.

The current view of the pathophysiology of T2D points to its compound nature, listing numerous factors as important components of the whole disease. Several groups of factors can be singled out, such as genetic [7], environmental [8], and dietary factors [9]. However, there are reports on the important role of the gut microbiome as a factor influencing the development, course, and progression of the disease [10]. The diversity of the microbiome in T2D can be affected both in terms of the number of reciprocal ratios of the different bacterial species that make up the intestinal microenvironment and in terms of quantity; observations as to the number of specific species indicate significance in both increasing and decreasing the number of individual individuals. In the reports cited, a reduction in the number of types such as *Bifidobacterium*, *Bacteroides*, *Faecalibacterium*, *Akkermansia*, and *Roseburia* stands out [10]. On the other hand, an increase in the number of types is postulated: *Ruminococcus*, *Fusobacterium*, and *Blautia* [10].

Demonstrating the important roles of GLP-1 analogs and GLP-1 receptor agonists in the modern therapy of type 2 diabetes and obesity, and given their complex pathophysiology, including potential changes in the gut microbiome environment, we undertook to examine the current state of knowledge on the effects of the cited drugs on the state of the gut microbiome. Moreover, the validity of the conducted research can be indirectly inferred by looking at analogous reviews made on a similar drug—metformin, where the relevance of conducting research on the effects of pharmacotherapy discernible in the disturbances of the qualitative and quantitative state of the intestinal microbiome was emphasized [11].

## 2. Materials and Methods

### 2.1. Literature Search

**Figure 1:**
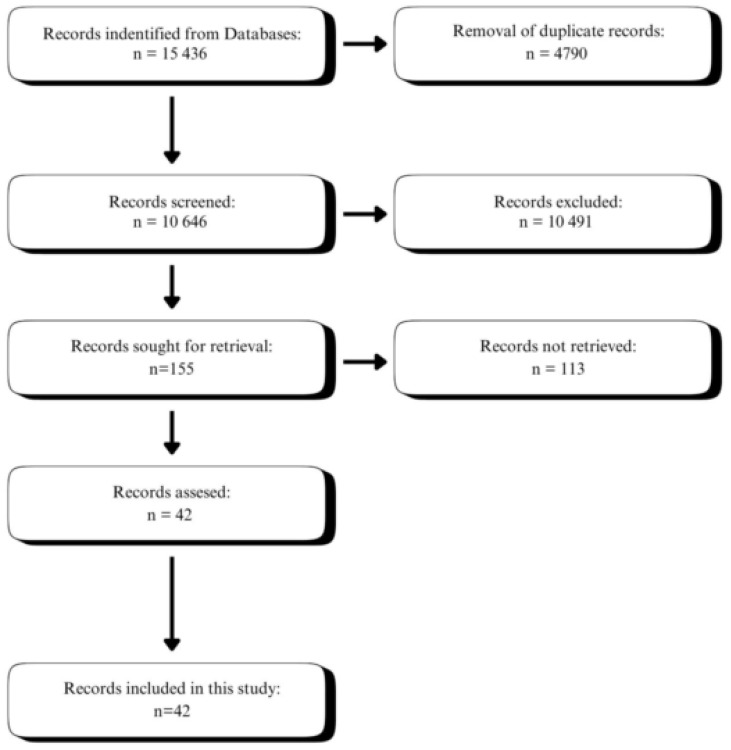
Search strategy.

Our search focused on the impact of GLP-1 agonists and analogues on the gut microbiome of both animals and humans, including those healthy and with accompanying conditions like obesity, polycystic ovarian syndrome, prediabetes, or diabetes. Our systematic search was conducted of PubMed and SCOPUS databases on 7 December 2024. The search strategy was shown in Figure 1. Initially, we searched the databases with the GLP-1 analogues, GLP-1 agonists, and GLP-1/GIP analogue. These keywords were then associated with microbiome, microbiota, bacteria, prebiotic, and probiotic. Additionally, we manually inspected the reference list to check for any publications of interest. We focused our search on observational studies and clinical trials, which is recommended for systematic reviews.

### 2.2. Study Selection Criteria

We followed the preferred reporting items for systematic reviews and meta-analyses (PRISMA) guidelines to ensure that our review was transparent as well as reliable [12]. We included only original studies that provide data on gut microbiota after GLP-1 analogue treatment, where the microbiota was analyzed using fecal samples. Additionally, we focused our search only on publications written in English.

### 2.3. Study Selection

We screened the abstracts of all articles to determine their eligibility. We have rejected all studies that showed the impact of the microbiome on the treatment outcome and not the impact of GLP-1 analogues on microbiota. Furthermore, we rejected the studies that did not state which type of GLP-1 analogue was used. With the help of an experienced librarian, we conducted a search of the Medline (from 2004 to 2025) and Cochrane Library (from 2004 to 2025) databases using terms such as GLP-1, combined with the term “microbiota”. In the next step, we included only records written in English and related to humans and mice and rats. In the last step, we excluded studies from the basic sciences or those containing duplicate data. The entire literature search procedure is detailed in Figure 1. The relevance and eligibility of retrieved records were considered by two authors independently. All discrepancies were resolved by consensus between the authors.

### 2.4. Data Collection Process

The data collection process was carried out using the Microsoft Excel program. This process was carried out by one author, while another reviewed it for reliability. The file included data about study characteristics, treatment, and participant information, and microbiome shift outcomes. All discrepancies were resolved by consensus between the authors.

### 2.5. Study Risk of Bias Assessment

Risk of bias was evaluated by the usage of Robins-I [13] and RoB2 [14] tools. This process was carried out by one author, while another reviewed it for reliability. All discrepancies were resolved by consensus between the authors.

### 2.6. Assessment Outcomes

Our primary goal in this systematic review was to investigate and analyze any changes at the phylum, genus, and species levels due to GLP-1 analogue administration. Furthermore, our secondary goal was to determine any variations in microbial richness and diversity due to GLP-1 agonist usage.

## 3. Results

### 3.1. Reviewed Studies

After screening, 155 records were identified, and after evaluation, we have retrieved 38 studies. In the final analysis, 9 human and 29 animal studies were included (Figure 1). Most of the studies included were performed in or after 2021. The studies varied in participants and used drugs as well as treatment duration. Out of 38 included studies, 28 were experimental studies (73.5%), 6 were longitudinal studies (15.5%), 2 were randomized trials (5%), 1 was a non-randomized trial (3%), and 1 was a spin-off study (3%).

### 3.2. Methods of Analysis for Gut Microbiota Composition in Studies

In this systematic review we have found that in all of the included studies fecal samples were analyzed to assess the microbial composition. 16S rRNA gene sequencing was the most commonly chosen method by researchers. Among various 16S rRNA gene sequencing methods, the V3–V4 regions were dominant, as shown in Table 1.

### 3.3. The Risk of Bias

**Figure 2:**
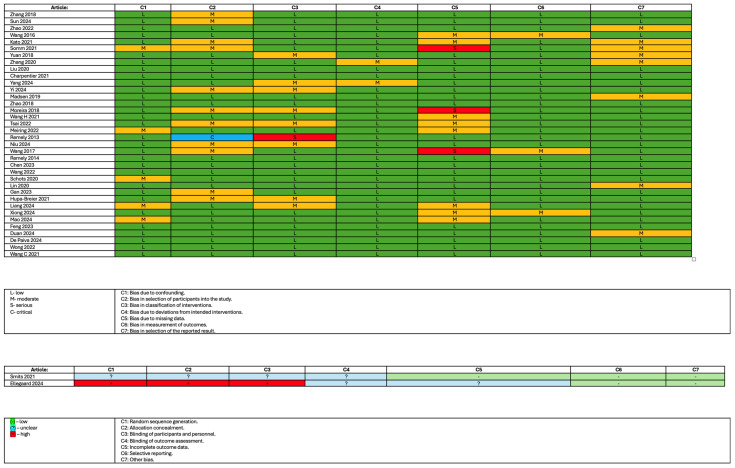
Risk of bias assessment [15,16,17,18,19,20,21,22,23,24,25,26,27,28,29,30,31,32,33,34,35,36,37,38,39,40,41,42,43,44,45,46,47,48,49,50,51,52].

In the studies included in this systematic review, one study had a high risk of bias in random sequence generation, allocation concealment, and blinding of participants and personnel while having an unclear risk of bias in blinding of outcome assessment and incomplete outcome data. One study had shown an unclear risk in four domains. Five studies had shown a serious risk of bias concern in one domain each, with one showing a crucial risk of bias in the selection of participants into the study with low or moderate risk in other domains. The risk of bias assessment is shown in Figure 2.

### 3.4. Changes in Bacterial Composition Resulting from GLP-1 Analogue Treatment

#### 3.4.1. Changes in Bacterial Genera and Species Induced by Liraglutide Treatment in the Animal Models

Among the 38 chosen studies, 27 focused on liraglutide. Of these, 8 were performed on human subjects, while 19 were carried out on animals (mice and rats). Animal studies clearly indicated that liraglutide influences the microbiome both on the phylum and genus, and at the species level as presented in Table 2.

The Bacteroidota phylum exhibited an increase in *Alistipes* [15,16] and *Butyricimonas* [17,18], both in two studies. The genus *Bacteroides* increased in five studies [16,19,20,21,22] and decreased in three [18,23,24]. *Norank_F_Bacteroidales_S24-7_Group* [23] and *Barnesiella* [24] both showed an increase in one study, while *Parabacteroides* increased in two studies [15,17] and decreased in one [23]. *Prevotella_9* [15,18] decreased in two studies.

In the Bacillota phylum, *Lactobacillus* increased in six studies with no reported decrease [16,17,18,22,24,25]. *Allobaculum* increased in four studies [17,18,20,22], and one study has reported its decrease [26]. *Clostridium* showed an increase in three studies [21,26,27], while two studies showed a decrease in its levels [18,27]. *Oscillospira* [17,26], *Blautia* [18,22], *Ruminococcus* [16,25], and *Turicibacter* [16,18] increased in two studies, while *Ruminococcus* [15,16,19,28] and *Turicibacter* [20] decreased in four and one, respectively. *Staphylococcus* [16], *Faecalibaculum* [16], *Candidatus Arthromitus* [18], and *Marvinbryantia* [18] decreased in one, while *Anaerotruncus* [23,27] and *Roseburia* [18,23] decreased in two. *Flavonifractor* [15,24] and *Lachnospiraceae_UCG-010* [15,23] both increased and decreased in one study. *Lachnoclostridium* [15], *Cellulosilyticum* [15], *Peptoniphilus* [15], and *Anaerostipes* [18] increased in one study while *Christensenellaceae_R-7_group* [15], *Oscillibacter* [23], *Ruminoclostridium_6* [15], and *Ruminiclostridium_9* [23] decreased in one. *Romboutsia* [23], *Sarcina* [26], *SMB53* [26], and *02d06* [26] all increased once, while *uncultured_f__Peptococcaceae* [23] and *Lachnospiraceae_NK4A136_group* [23] decreased once.

In the Pseudomonadota phylum, *Desulfovibrio* abundance increased in two studies [15,18] and decreased in one [23], while *Helicobacter* was reported to increase in one study [16] and decrease in two [25,29]. The genera *Escherichia* and *Shigella* both increased in one study [24], while *Desulfobacterota* [25] and *Klebsiella* [24] decreased in one. *Sutterella* [17], *Oxalobacter* [15], and *Sphingomonas* [15] all increased in one study.

In the Actinomycetota phylum, *Bifidobacteria* increased in two studies [15,21]. *Enterorhabdus* [15], *Gardnerella* [15], and *Johnsonella* [29] increased, and *Candidatus Saccharimonas* [23] decreased in one study.

In the Verrumicrobiota phylum, *Akkermansia* [16,23,27,29,30] increased in five studies, and none of the studies reported its decrease after liraglutide treatment in animals.

Finally, in the Fusobacteria phylum, one study reported an increase in the genus *Sneathia* [15].

At the species level in the Bacillota phylum, *Lactobacillus reuteri* [16], *Lactobacillus johnsonii* [16], *Anaerotruncus* sp. *G3* [27], *Anaerofustis*_*stercorihominis* [15], *Lactobacillus_mucosae* [15], *Ruminococcus_gravus* [15], and *Flavonifractor_plautii* [15] each increased in one study. In the Pseudomonadota phylum, increases were reported for *E. coli* [19], *Helicobacter typhi* [16], *Burkholderiales bacterium YL45* [27], *Brevundimonas vesicularis* [15], and *Moraxella_osloensis* [15], each in a single study. In the Bacteroidota phylum, *Bacteroides_acidifaciens* increased in one study [15]. *Akkermansia muciniphila*, representing the phylum Verrucomicrobiota, increased in 4 studies [17,27,29,30].

#### 3.4.2. Changes in Bacterial Genera and Species Induced by Liraglutide Treatment in Humans

Eight studies addressed the topic of gut microbiota in humans undergoing liraglutide treatment. Five studies reported no alterations in gut microbiota genera or species after treatment [31,32,33,34,35]. The results are shown in Table 3.

In the Bacillota phylum, *Lactococcus* [36] and an unknown genus in the family *Christensenellaceae* [37] were observed to increase in one study. Conversely, *Blautia* [36], *Dialister* [36], and *Megasphaera* [36] were shown to decrease in one study.

In the Bacteroidota phylum, only the genus *Alistipes* [38] was reported to decrease in one study. In the Pseudomonadota phylum, one study reported a decrease in *Sutterella* level [37]. In the Verrucomicrobiota phylum, *Akkermansia* increased in one study [37].

At the species level, *Faecalibacterium prausnitzii* [38] and *Peptostreptococcus anaerobius* [38], both belonging to the Bacillota phylum, increased in one study each. *Bacteroides vulgatus* [38], representing the Bacteroidota phylum, and *Akkermansia muciniphila* [38], representing the Verrucomicrobiota phylum, were both shown to increase in one study.

#### 3.4.3. Changes in Bacterial Genera and Species Induced by Exenatide or Exendin-4 in the Animal Model

Eight studies examined the effect of exenatide or exendin-4 on the composition of gut microbiota. Five studies investigated this impact using animal models. The results of our assessment are summarized in Table 4.

In the Bacteroidota phylum, a decrease in *Bacteroides* [24] was observed in one study, whereas increases in *Barnesiella* [24] and *Odoribacter* [24] levels were reported in one study each. In the Verrucomicrobiota phylum, an increase in *Akkermansia* was documented in one study [39]. Within the Actinomycetota phylum, *Enterorhabdus* [39] decreased in one study.

In the Bacillota phylum, *Streptococcus* [39,41] decreased across two studies. Additionally, *Romboutsia* [39], *Weissella* [39], *Marvinbryantia* [39], *Enterococcus* [39], *Flavonifractor* [24], and *Lactococcus* [41] each decreased in one study. *Ruminococcus* increased in one study [41]. *Lactobacillus* increased [24] in one and decreased in another [41].

At the species level in the Bacillota phylum, *Lactobacillus distasonis*, *Lactobacillus intestinalis*, and *Lactobacillus reuteri* increases were documented in one study [42]. Moreover, the Bacteroidota phylum showed an increase in *Alistipes finegoldii*, *Bacteroides acidifaciens*, *Bacteroides caccae*, *Parabacteroides distasonis*, *Parabacteroides goldsteinii*, and *Parabacteroides merdae*, all in one study [42]. Finally, in the Verrucomicrobiota phylum, an increase in *Akkermansia muciniphila* was noted in one study [42].

#### 3.4.4. Changes in Bacterial Genera and Species Induced by Exenatide in Humans

Three studies reported changes over the exenatide treatment in the microbiome. The results are summarized in Table 5.

In the Bacillota phylum, *Coprococcus* increased in one study [43] while *Anaerotignum*, *Lactococcus*, *Flavonifractor*, and *Oscillospira* decreased in the same study [43]. In the Bacteroidota phylum, it was reported that only *Prevotella* increased once [43]. In the Actinomycetota phylum, *Bifidobacterium* increased in one study [43].

At the species level, an increase in *Bacteroides uniformis* [43] and *Phocaeicola vulgatus* [43], belonging to the Bacteroidota phylum, has been seen in one study each. In the Bacillota phylum, *Hungatella hathewayi*, *Lachnospira eligens*, *Roseburia hominis*, and *Roseburia intestinalis* increased in one study [43]. In the Verrucomicrobiota phylum, *Akkermansia muciniphila* increased in one study [38].

#### 3.4.5. Changes in Bacterial Genera and Species Induced by Dulaglutide Treatment in an Animal Model

Only one study reported changes in the intestinal microbiota over dulaglutide treatment in animals. This study outcome is shown in Table 6.

In the Bacillota phylum, dulaglutide treatment was associated with an increase in *Aerococcus*, *Clostridium sensu stricto*, *Clostridium XlVb*, and *Enterococcus* in one study. Moreover, increases in *Ruminococcus*, *Pseudoflavonifractor*, *Lachnospiracea_ incertae_sedis*, and *Oscillibacter* were reported while *Coprobacillus*, *Macrococcus*, *Roseburia*, and *Streptococcus* showed decreases, all in one study [44].

After Dulaglutide treatment, it was reported that in the Bacteroidota phylum, *Bacteroides*, *Parabacteroides*, *Barnesiella*, and *Alistipes* all increased in one study [44].

In the Pseudomonadota phylum, increases in *Escherichia*, *Shigella*, and the genus *Parasutterella* were observed in one study [44]. In the Verrucomicrobiota phylum, *Akkermansia* also increased in one study [44].

At the species level, this study did not report any changes.

#### 3.4.6. Changes in Bacterial Genera and Species Induced by Dulaglutide Treatment in Humans

Only one study showed changes in the intestinal microbiota over dulaglutide treatment in humans. This study outcome is shown in Table 7.

In the Bacteroidota phylum, *Bacteroides* was noted to increase while *Prevotella* both increased and decreased over the study period [45].

In the Bacillota phylum, it was reported that *Lactobacillus* increased while *Blautia* and *Ruminococcus* showed a decrease [45].

Within the Actinomycetota phylum, *Bifidobacterium* both increased and decreased over the study period [45].

At the species level, the study did not report any changes.

#### 3.4.7. Changes in Bacterial Genera and Species Induced by Semaglutide Treatment in the Animal Model

Six studies reporting changes in the gut microbiota after semaglutide treatment were included in this review. We found no studies researching the impact of semaglutide treatment on the gut microbiome in humans. The outcome of our assessment is shown in Table 8.

In the Bacteroidota phylum, *Bacteroides* [46], *Alistipes* [47], *and Alloprevotella* [47] increased in one study while *Odoribacter* decreased to the same extent [48].

In the Bacillota phylum, *Allobaculum* [49], *Blautia* [50], *Enterococcus* [51], and *Clostridia _UCG_014* [48] were demonstrated to increase in one study each, while *Ligilactobacillus* [47], *Lachnospiraceae_NK4A136_group* [49], and *unclassified_f_Oscillospiraceae* [49] showed a decrease in one study. *Dubosiella* increased in two studies [49,51] and decreased in one [48], while *Lactobacillus* increased [48] and decreased in one study [47]. The *Romboutsia* genus was reported to be decreased in two studies [48,51].

In the Pseudomonadota phylum, it was noted that *Escherichia* [51], *Shigella* [51], and *Helicobacter* [46] were shown to increase in one study.

In the Verrucomicrobiota phylum, *Akkermansia* was observed to increase in two studies [48,49].

In the Actinomycetota phylum, *Coriobacteriaceae UCG-002* was reported to show an increase in two studies [48,51].

At the species level in the Bacteroidota phylum, *Alistipes muris* [46] increased and *Muribaculaceae bacterium Isolate-037* [46] decreased in one study each. *Bacteroides acidifaciens* increased in one study and decreased in another [46,49]. In the Bacillota phylum, *Blautia coccoides* [50] increased and *Lepagella muris* [46] decreased in one study.

### 3.5. Effects of GLP-1 Analogues on Gut Microbiota Diversity

#### 3.5.1. Effects of Liraglutide Treatment on Gut Microbiota Diversity

In high-fat diet-fed Wang C et al. [52] reported an increase in microbiota diversity by ACE, Chao, Simpson, Shannon, and Sobs indices. All of the indices, except the Simpson index, which remained stable, have shown an increase after both low and high dose liraglutide treatment (200 and 400 µg/kg/d) when compared to HFD-fed mice without treatment. This showed an increase in microbial diversity with liraglutide treatment, especially observed with low-dose liraglutide application. Moreover, Zhao L et al. [17] also reported that Chao1, Shannon’s, and Simpson’s indices increased with liraglutide usage when compared to HFD-fed mice. Furthermore, the Sobs index used by Moreira GV et al. [29] to investigate the changes in phylogenetic microbial diversity showed an increase after liraglutide treatment. Madsen MSA et al. [27] stated that the richness in microbiota tended to increase after liraglutide usage, but it was not statistically significant. Finally, Charpentier J et al. [24] reported no statistically significant changes in Shannon’s index, indicating no change in microbial diversity.

When examining streptozotocin-induced diabetic mice, Yang Q et al. [25] reported no changes in the diversity of the gut microbiota after checking the Chao1 index. Conversely, Liu Q et al. [23] declared a significant decrease in alpha diversity even though the Sobs index was not affected in diabetic mice. Wang L et al. [18] presented a decrease in diversity based on Shannon’s and Simpson’s indices in normoglycemic mice, while in hyperglycemic mice, it was restored to a normal level after treatment.

In other mouse studies, Somm E et al. [20] did not report any changes in microbial diversity in MCD-fed mice. In dehydroepiandrosterone-induced polycystic ovary syndrome (PCOS) mice, Xiong C et al. [46] reported no change in beta diversity evaluated with principal coordinate analysis (PCoA). Wang H et al. [30] in the study with lipid-induced hepatic stenosis mice reported that Chao1 and Shannon’s indices increased and did not show significant change, respectively.

Three studies investigated diabetic rats. Zhang Q et al. [15] stated a decreased alpha diversity using Chao1 and Shannon’s indices and lowered beta diversity, evaluated by PCoA. A decrease in total bacterial number was also shown by Yuan X et al. [21] with 16S-based PCR and DEEP analysis. 16S rRNA V3–V4 region sequencing performed by Zhao L et al. [28] showed a significant decrease in Chao and Shannon indices, indicating a lowered microbiota richness.

In HFD-fed rats, Yi B et al. [26] found no change in Chao1 and ACE indices; however, they found a change in the Simpson index, which was upregulated with treatment. Zhang N et al. [22] reported no changes in diversity with Simpson and Shannon indices, while they showed a decrease in microbial richness with Chao1 and ACE indices.

Out of eight human studies investigating the impact of GLP-1 analogues, seven focus on patients with type 2 diabetes mellitus. Remely M et al. [34] found no differences in microbiome diversity in T2DM over the 4-month intervention. Meiring S et al. [33] reported no difference in either alpha or beta diversity of T2DM participants over a 3-month study period. Same results about alpha and beta diversities were reported by Smits MM et al. [31] with Shannon’s index, PCoA, and multilevel principal component analysis (PCA). To the same conclusion about alpha diversity came Tsai CY et al. [32]; on the other hand, they found statistically significant changes in beta diversity when comparing GLP-1 agonist responders and non-responders with PCoA. In the combined treatment of liraglutide and metformin, over the 42-day study period, Wang Z et al. [37] found no diversity differences to participants who used only metformin treatment. Moreover, Niu X et al. [36] tested Shannon, Simpson, and Chao1 indices between the control, metformin, and metformin + liraglutide groups and came to the conclusion that liraglutide partially improved microbiota diversity, seen by a change in Shannon’s and Simpson’s indices, but the Chao1 index still pointed to a higher diversity in the control group. Furthermore, although liraglutide lowered beta diversity, it changed it to be more similar to healthy individuals. Finally, Remely M et al. [38] stated no differences in microbial abundance after liraglutide intervention.

The last out of 8 human studies that looked into the impact of liraglutide treatment focused on patients with bile acid diarrhea, in which Ellegaard AM et al. [35] found no significant differences in microbial diversity with both Chao1 and Shannon’s indices.

#### 3.5.2. Effects of Exenatide/Exentin-4 Treatment on Gut Microbiota Diversity

During the study conducted on high-fat diet (HFD)-fed mice, Charpentier J et al. [24], using a Shannon index of taxonomy, did not find any changes in microbial diversity. Similar results in HFD-fed mice have been obtained by Schots PC et al. [41]; they did not observe any statistically important changes in alpha diversity of the microbiome. Finally, Lin K et al. [42] also reported no difference in alpha diversity using abundance-based coverage estimator (ACE) but found an increase in beta diversity.

Chen Y et al. [39] investigated the impact of exenatide on gut microbiota in diabetic mice, using Shannon and Simpson indices. Neither of the indices showed any statistically significant difference while exenatide affected the beta diversity.

In patients with both obesity and polycystic ovarian syndrome (PCOS), Gan J et al. [43] announced that metformin plus exenatide therapy hugely impacted intestinal microbiota composition, which was investigated by PCoA.

Remely M et al. [34], in a study performed on humans with type 2 diabetes, reported no changes in microbial abundance.

#### 3.5.3. Effects of Dulaglutide Treatment on Gut Microbiota Diversity

Hupa-Breier KL et al. [44] examined the non-diabetic mouse model of NASH. Both Shannon and Simpson indices were increased after dulaglutide treatment, indicating increased alpha diversity. They also reported an increase in beta diversity when compared to the mice treated with saline vehicle.

In type 2 diabetic humans, Liang L et al. [45] found no changes in microbiota diversity and abundance after 1 week of dulaglutide treatment but reported changes in Chao1 and ACE, whose levels were lower than before treatment, with stable Simpson and Shannon indices. This led to a conclusion that dulaglutide treatment impacts the abundance but not the diversity of microbiota.

#### 3.5.4. Effects of Semaglutide Treatment on Gut Microbiota Diversity

In Type 2 diabetes mellitus mice, de Paiva IHR et al. [50] reported changes in beta diversity but also in species diversity and abundance. The same result regarding beta diversity using PCoA was received by Mao T et al. [47]. Moreover, they obtained an increase in alpha diversity after semaglutide treatment using 16S rRNA sequencing.

Duan X et al. [49] reported changes in Shannon, Chao, ACE, and Sobs indices in HFD-fed mice. In this study, semaglutide upregulated the microbial diversity. On the other hand, it did not affect the Simpson index. On the contrary, Feng J et al. [48], after examining Chao1, Shannon, Simpson, and ACE indices, found an increase only in the ACE index. They also reported a significant change in bacterial diversity with PCoA plots.

Finally, Xiong C et al. [46] tested the Dehydroepiandrosterone-Induced Polycystic Ovary Syndrome Mice. Both ACE and Shannon indices were decreased after semaglutide treatment, indicating a downregulation of semaglutide on microbiota richness and diversity.

### 3.6. Effects of GLP-1 Receptor Agonists on Metabolic Syndrome

Semaglutide, liraglutide, exenatide, or dulaglutide, which belong to the GLP-1 receptor agonists, are broadly used in type II diabetes mellitus and obesity treatment. They treat these illnesses by increasing insulin secretion as a result of food intake. Moreover, they suppress the glucagon release and gastric emptying, which finally leads to better glycemic control and body mass reduction. More and more studies suggest that GLP-1 agonists effectiveness in mitigating metabolic syndrome can be a result of microbiome transformation. It has been shown that beneficial bacteria such as *Akkermansia muciniphila* or *Bacteroides*, associated with better metabolic homeostasis, have thrived after GLP-1 receptor agonist administration. They also negatively correlate with the inflammatory state of the intestines. Furthermore, microbial modulation by GLP-1 agonists can result in an increase in short chain fatty acids. Butyrate, propionate, and acetate improve insulin sensitivity, which is crucial in glucose metabolism. GLP-1RAs can also reduce the abundance of Bacillota highly associated with obesity and insulin resistance. Apart from Bacillota, they can also reduce lipopolysaccharide-producing bacteria, resulting in a lower level of LPS, which can explain the positive effect of GLP-1RAs in metabolic syndrome. Decreased inflammatory state can also protect pancreatic β-cells. Moreover, it can be beneficial in inflammatory bowel diseases such as Crohn’s disease or ulcerative colitis.

## 4. Discussion

GLP-1 was discovered in 1984 by Svetlana Mosjov. The first GLP-1 agonist (exenatide) was approved in 2005, and the popularity of this group of drugs has only grown ever since. Thanks to better glycemic control, weight loss, and cardiovascular benefits, GLP-1 agonists are now considered to be a standard in T2D therapy. It is known that the microbiome plays a vital role in human health [53,54,55]. Therefore, we performed a systematic review to analyze the current knowledge on the impact of GLP-1 analogues on the intestinal microbiome.

Our findings of this analysis supply precious insights into the interplay between pharmacological interventions and gut microbiota. We provide insights into the impact of GLP-1 analogues on the microbiome diversity and composition. The complexity of the gut microbiome is highlighted by the differences across studies, which vary in treatment methods, bacterial taxa, as well as conditions of the experiments.

Liraglutide, which was the most studied GLP-1 receptor agonist, treatment changes showed significant alternations in microbiome composition. We showed increased abundances of *Alistipes* and *Butyricimonas* genera from the Bacteroidota phylum. Moreover, in the Bacillota phylum, *Lactobacillus* and *Allobaculum* increased. From these results we can conclude that liraglutide administration promotes the growth of genera relevant to beneficial metabolic functions. It is worth noticing that the species *Akkermansia muciniphila* increased, as aligned with improved intestinal barrier integrity and metabolic health [10]. The change towards a healthier microbiome profile is also shown by a shift in *Ruminococcus* and *Turicibacter,* known for their involvement in intestinal inflammation [56,57].

Exenatide and exendin-4 administration showed various effects on the microbiome composition in animal and human studies. In animal studies it was reported that *Akkermansia, Barnesiella*, and *Ruminococcus* genera all increased upon exenatide or exendin-4 treatment, while the genera associated with dysbiosis, such as *Streptococcus* and *Marvinbryantia*, decreased [58,59]. Human studies obtained fewer changes. *Coprococcus* and *Bifidobacterium* genera, associated with improved metabolic and inflammatory profiles, increased. This may suggest a potentially differential impact of exenatide due to host-specific factors.

Dulaglutide effects were studied not as extensively as other GLP-1 analogues; however, they showed a promising trend. In animal studies it was reported that dulaglutide was significantly associated with increases in *Bacteroides*, *Akkermansia*, and *Ruminococcus*, genera connected to an improved metabolic model. Changes in humans after dulaglutide treatment demonstrated limited changes. Worth noticing was an increase in *Lactobacillus* genera. This suggests a potentially positive impact of dulaglutide on the gut microbiome.

Semaglutide demonstrated significant alterations in the microbiome in animals. An increase in *Alistipes*, *Alloprevotella*, and *Akkermansia* genera could not go without notice, as they associated with a healthier metabolic model. An increase in *A. muciphila* known for its positive metabolic effects on human health support semaglutide’s potential as a therapeutic. On the other hand, some studies showed results suggesting decreased diversity indices. This may indicate that semaglutide’s effects vary over the metabolic state, diet and concomitant diseases.

Many studies reported the impact of microbiota on inflammation and insulin resistance [60,61]. The *Akkermansia* genus was increased after all GLP-1 analogue administrations. The genus includes the species *Akkermansia muciniphila,* which is associated with lower cardiovascular risk, improved weight management, and intestinal health [62]. Moreover, the *Faecalibacterium* and *Lactobacillus* genus were increased in several studies, and they contain several species with a positive impact on human health. We want to highlight the *Lactobacillus reuteri* species, which was elevated after liraglutide and exenatide administration. *L. reuteri* positively impacts GLP-1 secretion, intestinal barrier, and anti-inflammatory functions [63,64,65]. Furthermore, *Faecalibacterium prausnitzii* levels were also increased after liraglutide treatment. It is a known butyrate producer with anti-inflammatory effects [66]. This leads to a conclusion that microbial changes may impact T2D pathogenesis, as well as treatment outcomes.

Accumulating evidence indicates that glucagon-like peptide-1 receptor agonists (GLP-1RAs) may induce compositional shifts in the gut microbiota, though significant heterogeneity across studies complicates interpretation. Variability in study design (e.g., randomized controlled trials vs. observational cohorts), intervention duration (8–52 weeks), and analytical methodologies (16S rRNA sequencing vs. metagenomic sequencing) contributes to conflicting reports, such as inconsistent increases in *Akkermansia muciniphila* or ambiguous changes in microbial alpha diversity. Furthermore, confounding variables—including dietary intake, concomitant antidiabetic medications (e.g., metformin), and lifestyle factors—are frequently unaccounted for, obscuring the direct contribution of GLP-1RAs to observed microbiota alterations.

Preclinical models propose mechanistic pathways linking GLP-1RA-induced microbiota changes to metabolic improvements, such as enhanced short-chain fatty acid production or attenuation of endotoxemia. However, human data remain largely associative, with correlations between specific microbial taxa (e.g., Roseburia, Faecalibacterium) and metabolic parameters (e.g., HbA1c reduction, weight loss) failing to establish causality. The predominance of short-term, uncontrolled studies further limits insight into whether microbial shifts persist or drive clinically meaningful outcomes.

To address these gaps, future investigations should employ longitudinal cohort studies with standardized microbiota profiling, rigorous adjustment for confounders, and integration of multi-omics approaches (metagenomics, metabolomics) to delineate host-microbe interactions. Until such evidence is available, assertions of clinically significant microbiota modulation by GLP-1RAs should be circumspect, acknowledging that observed changes may represent secondary effects of metabolic improvement rather than primary therapeutic mechanisms. Current findings underscore the need for cautious interpretation, framing gut microbiota modulation as a plausible but unproven contributor to the pleiotropic effects of GLP-1RA therapy.

We need to highlight the importance of further studies, including human in vivo studies. Out of 38 studies, 9 investigated the impact of GLP-1 analogues in humans, while 29 were conducted on animals. The number of studies concerning liraglutide, exenatide or exentin-4, dulaglutide, and semaglutide in animals was nineteen, eight, one, and six, respectively. The results obtained in the animal model should be confirmed in further studies in humans. The number of studies regarding liraglutide, exenatide, and dulaglutide in humans was eight, three, and one, in order. To better understand the impact of GLP-1 receptor agonists on the microbiome and their application in T2D treatment, larger sample sizes are required.

Finally, in our systematic review we provide many examples of GLP-1 analogues’ impact on the microbiota. However, further studies on bacterial involvement in T2D are crucial to explain the impact of bacteria and to investigate potential therapeutics.

## 5. Limitations

The aim of this publication is to provide a comprehensive evaluation of the impact of GLP-1 agonists on the gut microbiome. However, we need to keep in mind various limitations. Firstly, the included studies showed a high diversity in the number and concomitant diseases of participants, the GLP-1 analogue dosage, sequencing methods, as well as the duration of drug administration. Moreover, the risk of bias assessment revealed varying levels across studies. While it was minimized in most studies, some still showed a higher risk of bias, which may have altered the overall conclusions. This analysis focused on changes at the bacterial genus and species levels, which is suboptimal, as it overlooks potential discrepancies at the strain level. Furthermore, many studies have had confounding factors that could have altered the results, such as the type of disease, treatment duration, and multi-drug therapy. Most of the studies were published in or after 2021, and the duration of GLP-1 usage was relatively short. This may hinder our ability to understand the long-term effects of using GLP-1 analogues. Finally, we need to underline that the bacterial taxonomy is very variable. This may lead to many inaccuracies. All of the above-said limitations could have altered obtained results, which limits the ability to acquire unambiguous conclusions.

## 6. Summary and Conclusions

In our systematic review, based on the PRISMA guidelines, we focused on gathering the knowledge of the effects of GLP-1 analogue medications on gut microbiota richness, composition, and abundance in both animals and humans. Our primary goal was to investigate the changes of bacteria following GLP-1 analogue administration on the phylum, genus, and species level. Moreover, as a secondary goal, we determined any alternations in richness and diversity. We can conclude, following the obtained research results of our study, that liraglutide promotes the growth of beneficial genera relevant for beneficial metabolic functions. Exenatide and exendin-4 administration showed various effects on the microbiome composition in animal and human studies. In animal models, it increased genera associated with improved metabolism; however, in human models, genera linked to better metabolic functions and escalated inflammation increased. Following dulaglutide administration, increases in *Bacteroides*, *Akkermansia*, and *Ruminococcus*, genera connected to an improved metabolic model, were significant. Finally, varied results were obtained after semaglutide treatment, in which *A. muciniphila*, known for its positive metabolic functions, increased; however, microbial diversity decreased.

The effect of GLP-1 analogues on the gut microbiota may have a positive impact on the long-term treatment of diabetes, preventing complications of this disease and maintaining a proper body weight. Further research is essential to confirm the above findings and to better recognize their implications for the clinical outcomes of patients.

## Figures and Tables

**Table 1:** **Description of the included studies**.

No.	Article	Type of Study	Description of the Experiment and Study Groups/Description of the Study	Method of Analysis	Conclusion
1	Zhang 2018 [15]	Experimental	Diabetic male rats	16S rRNA V3-V4	In liraglutide-treated diabetic male rats, short-chain fatty acid (SCFA)-producing bacteria, including Bacteroides, Lachnospiraceae, and the probiotic Bifidobacterium, were selectively enhanced. Lactobacillus showed a negative correlation with fasting blood glucose.
2	Sun 2024 [16]	Experimental	Wild-type and Rag1−/−, Rag2−/− i, Rorcgfp/gfp mice	16S rRNA V3-V4	Liraglutide administration significantly alleviates the DSS-induced colitis symptoms and ameliorates histological damage.
3	Zhao 2022 [17]	Experimental	C57BL/6 mice	16S rDNA V3-V4	Correlation analysis showed that TC and LDL were linked to harmful bacteria and inversely related to beneficial ones. Liraglutide improves dyslipidemia in HFD-fed mice by modulating gut microbiota, especially ***Akkermansia***, suggesting this as a key mechanism of its effect.
4	Wang 2016 [18]	Experimental	ApoE−/− mice on C57BL/6 background	16s rDNA V1-V3	The GLP-1 receptor agonist liraglutide modulates gut microbiota composition, promoting a lean-associated profile that aligns with its weight-loss effects.
5	Kato 2021 [19]	Experimental	Mice	16S/18S rRNA	Increased levels of caseinolytic protease B, a component of Escherichia coli, and norepinephrine were observed in the cecum.
6	Somm 2021 [20]	Experimental	Mice fed a regular diet or a methionine-choline deficient (MCD) diet.	16S rDNA V3-V4	Liraglutide altered gut microbiota composition affected by the MCD diet, restoring normal ***Bacteroides*** levels and shifting ***Erysipelotrichaceae*** from ***Allobaculum*** to ***Turicibacter*.**
7	Yuan 2018 [21]	Experimental	*STZ*-induced Sprague-Dawley rats	16S rRNA V3	GLP-1, by restoring the intestinal flora dysbiosis, can moderate STZ-induced DM treatment.
8	Zhang 2020 [22]	Experimental	Male rats fed a high-fat diet. After 12 weeks, two rats	16s rDNA V4-V5	Liraglutide significantly modified the structure and composition of HFD-disrupted gut microbiota in comparison to rats on a normal diet.
9	Liu 2020 [23]	Experimental	Male db/db mice	16S rRNA V3-V4	Bacteria like ***Parabacteroides*, *Oscillibacter*,** and ***Prevotellaceae*_*UCG*-*001*** produce anti-inflammatory SCFAs and influence T cell differentiation. In the LG group, bacteria such as ***Anaerotruncus*** and ***Lachnospiraceae*** were reduced and correlated with ALT and AST. Oscillibacter and Klebsiella correlated with ALT, while Desulfovibrio and Bacteroides correlated with AST.
10	Charpentier 2021 [24]	Experimental	Diet-induced dysmetabolic mice	16S rRNA	Data showed that GLP-1 receptor agonists, like liraglutide, regulate insulin secretion by simultaneously modulating intestinal immunity and bacterial ecology.
11	Yang 2024 [25]	Experimental	Mouse diabetic model	16S rRNA	Empagliflozin reduced the ***Firmicutes*/*Bacteroidota*** ratio, while liraglutide had no effect. Both decreased ***Helicobacter*** and increased Lactobacillus. Empagliflozin also raised ***Muribaculaceae*, *Muribaculum*, *Olsenella*, and *Odoribacter***, whereas liraglutide increased ***Ruminococcus***.
12	Yi 2024 [26]	Experimental	Diabetic kidney disease rats	16S rRNA V3-V4	The gut microbiota-metabolites-kidney axis, particularly ***Clostridium*-*5*-*OP*-*ELD***, may serve as a potential target of Liraglutide in DKD.
13	Madsen 2019 [27]	Experimental	Diet-induced obese mice.	16S rRNA V3-V4	DIO mice showed similar gut bacterial changes after liraglutide and GUB09-145 treatment, with shifts in low-abundant species and metabolic pathways.
14	Zhao 2018 [28]	Experimental	Wistar and Goto–Kakizaki (GK) rats	16S rDNA V3-V4	Liraglutide prevents weight gain by altering gut microbiota, reducing diversity, increasing the ***Firmicutes*/*Bacteroidetes*** ratio, and promoting lean-associated microbial profiles in obesity and T2DM.
15	Moreira 2018 [29]	Experimental	C57BL/6J mice	16S rRNA V3-V4	Liraglutide modified gut microbiota by reducing ***Proteobacteria*** and increasing ***Akkermansia muciniphila***, contributing to weight loss, microbiota balance, and NAFLD improvement.
16	Wang H 2021 [30]	Experimental	HIF-2α heterozygous knockout (HIF-2α+/−) and wild-type (WT) littermate mice	16S rRNA V3-V4	HIF-2α is essential for the effect of liraglutide in promoting the increased abundance of ***Akkermansia muciniphila***.
17	Smits 2021 [31]	Randomized trial	Fifty-one adults (humans) with type 2 diabetes	16S rRNA V3-V4	Liraglutide and sitagliptin improve glucose metabolism, body weight, and bile acids as add-ons to metformin or sulphonylureas, independent of changes in intestinal microbiota.
18	Tsai 2022 [32]	Longitudinal study	Fifty-two adults	16S rRNA	***Bacteroides dorei***, ***Lachnoclostridium* sp**., and ***Mitsuokella multacida*** were significant after adjusting for baseline glycohemoglobin and C-peptide concentrations, two clinical confounders.
19	Meiring 2022 [33]	spin-off study	Fecal samples from 16 patients were collected for Illumina shotgun sequencing at baseline and three months post-DMR.	shotgun sequencing	Gut microbiota diversity changes correlate with HbA1c, PDFF, and metabolic improvements after DMR and GLP-1 receptor agonist treatment in type 2 diabetes.
20	Remely 2013 [34]	Longitudinal study	Fourteen obese individuals (OC) with no established insulin resistance and twenty-four insulin-dependent type 2 diabetes (D) patients	16S rRNA	Gut microbiota in obesity and type 2 diabetes may influence FFAR epigenetics, affecting satiety and hunger. Butyrate supports regenerative medicine by promoting epigenetic remodeling and gene expression.
21	Ellegaard 2024 [35]	Randomized trial	Fifty-two adults	16S rRNA	Neither treatment resulted in changes to the composition of fecal microbiota.
22	Niu 2024 [36]	Longitudinal study	All 32 participants in the pre-diabetic/overweight control group (NCP) later transitioned to the untreated T2DM group (UNT) upon diagnosis.	16S rRNA V4	MET and MET + LRG treatment significantly altered gut microbiota from T2DM diagnosis, with MET + LRG showing distinct changes, suggesting LRG’s additive effect.
23	Wang 2017 [37]	Longitudinal study	37 patients with T2DM	16S rRNA V4	The study suggests that altering the gut microbiome may play a key role in the mechanisms of action of metformin and liraglutide.
24	Remely 2014 [38]	Longitudinal study	T2DM patients	16S rRNA	Gut microbiota composition varies between groups with GLP-1 analogue therapy and that with no therapy.
25	Chen 2023 [39]	Experimental	C57BL/6J mice	16S rDNA V3-V4	Exenatide reduced pathogenic bacteria, increased ***Akkermansia***, improved testosterone, and lowered inflammation. Fecal bacteria from the Exe group reduced harmful microbes and protected against testicular damage in diabetic mice.
26	Wang 2022 [40]	Experimental	Hypertensive rats (SHR). SHR and Wistar rats.	16S rRNA V3-V4	The treatments corrected dysbiosis in SHR rats by reducing ***Porphyromonadaceae*** and increasing ***Lactobacillus***. GLP-1 improved blood pressure and cardiomegaly by restoring the gut microbiome and reducing ventricular hypertrophy.
27	Schots 2020 [41]	Experimental	57bl/6J mice	16s rDNA V4-V5	Exenatide prevented the HFD-induced increase in ***Lactococcus*** and caused a decrease in the abundance of ***Streptococcus*** compared to the HFD group.
28	Lin 2020 [42]	Experimental	Mice	16S/18S rRNA	***Lactobacillus reuteri*** increased in bacterial counts, while ceramide levels in mouse serum decreased, with a negative correlation between *L. reuteri* and ceramide. The decrease in ceramide was linked to overexpression of alkaline ceramidase 2 (Acer2). *L. reuteri* may work synergistically with GLP-1 RA for therapeutic benefits.
29	Gan 2023 [43]	Non-randomized controlled trial	Obese patients with polycystic ovary syndrome (PCOS)	Paired-end sequencing	Both groups had high levels of ***Firmicutes*, *Bacteroidetes*, *Uroviricota***, ***Actinobacteria*, and *Proteobacteria***. Post-treatment, probiotics like ***Phocaeicola*** and ***Anaerobutyricum*** increased. ***Clostridium***, ***Fusobacterium***, and ***Oxalobacter*** dominated in the post-MF group, while ***Lactococcus garvieae*, *Clostridium perfringens*, and *Coprococcus* sp. *AF16_5*** were dominant in the post-COM group.
30	Hupa-Breier 2021 [44]	Experimental	C57BL/6HanJ Ztm. mice	real-time PCR	The study highlights dulaglutide’s significant anti-inflammatory effects, especially when combined with empagliflozin, by modulating pro-inflammatory immune responses and microbiome dysbiosis.
31	Liang 2024 [45]	Longitudinal study	Patients with newly diagnosed T2DM with no GLP-1RA history.	16S rRNA	No significant changes in gut microbiota after 1 week of dulaglutide treatment in newly diagnosed T2DM patients. After 48 weeks, notable changes occurred, including a significant reduction in microbial abundance.
32	Xiong 2024 [46]	Experimental	Dehydroepiandrosterone (DHEA)-induced PCOS mice	shotgun sequencing	The greater efficacy in weight loss compared with liraglutide observed after semaglutide intervention was positively related with ***Helicobacter***.
33	Mao 2024 [47]	Experimental	db/m and db/db mice	16S rRNA	Significant changes in ***Alloprevotella*, *Alistipes*, *Ligilactobacillus*, and *Lactobacillus*** were observed between the db/db control group and the semaglutide treatment group. LDA scores showed increased ***Alloprevotella* and *Alistipes*, while *Ligilactobacillus* and *Lactobacillu****s* decreased after semaglutide treatment.
34	Feng 2023 [48]	Experimental	C57BL/6J mice	16S rRNA V3-V4	Semaglutide may influence gut microbiota composition and structure, impacting cognitive function and inflammation.
35	Duan 2024 [49]	Experimental	Mice fed a high-fat diet	16S rRNA	Semaglutide could alter gut microbiota dysbiosis, which may explain the anti-obesity effects of semaglutide.
36	De Paiva 2024 [50]	Experimental	C57BL/6 mice	16S rRNA	Semaglutide may treat depression and anxiety by reducing hippocampal neuroinflammation and promoting neurogenesis through the insulin/GLP-1 pathway and gut microbiota modulation.
37	Wong 2022 [51]	Experimental	Rag2−/−;Il2rg−/− mice and Glp1rTcell−/− mice	16S rRNA V3-V4	The intestinal intraepithelial lymphocyte GLP-1R is required for actions of GLP-1 on gut microbiota and for selective restraint of local and systemic T cell-induced, but not lipopolysaccharide-induced, inflammation.
38	Wang C 2021 [52]	Experimental	Mice with a high-fat diet	16S rRNA	Liraglutide increases ***Lactobacillaceae*** abundance in hyperlipidemic mice, enhances LAB bile tolerance by upregulating bile salt hydrolases, and liraglutide-sensitive LAB lysates directly downregulate HMGCR.

**Table 2:** Impact of liraglutide treatment on gut microbiota in animals.

Phylum	Genus	Increased	Decreased
Bacteroidota	*Bacteroides*	5	3
Bacteroidota	*Alistipes*	2	0
Bacteroidota	*Parabacteroides*	2	1
Bacteroidota	*Butyricimonas*	2	0
Bacteroidota	*Prevotella_9*	0	2
Bacteroidota	*Norank_F_Bacteroidales_S24-7_Group*	1	0
Bacteroidota	*Barnesiella*	1	0
Bacillota	*Ruminicoccus*	2	4
Bacillota	*Lactobacillus*	6	0
Bacillota	*Turicibacter*	2	1
Bacillota	*Staphylococcus*	0	1
Bacillota	*Faecalibaculum*	0	1
Bacillota	*Allobaculum*	4	1
Bacillota	*Oscillospira*	2	0
Bacillota	*Clostridium*	3	2
Bacillota	*Anaerotruncus*	0	2
Bacillota	*Anaerostipes*	1	0
Bacillota	*Blautia*	2	0
Bacillota	*Candidatus Arthromitus*	0	1
Bacillota	*Roseburia*	0	2
Bacillota	*Marvinbryantia*	0	1
Bacillota	*Flavonifractor*	1	1
Bacillota	*Lachnoclostridium*	1	0
Bacillota	*Cellulosilyticum*	1	0
Bacillota	*Christensenellaceae_R-7_group*	0	1
Bacillota	*Lachnospiraceae_UCG-010*	1	1
Bacillota	*Peptoniphilus*	1	0
Bacillota	*Ruminoclostridium_6*	0	2
Bacillota	*Romboutsia*	1	0
Bacillota	*Lachnospiraceae_NK4A136_group*	0	1
Bacillota	*Oscillibacter*	0	1
Bacillota	*Ruminiclostridium_9*	0	1
Bacillota	*uncultured_f__Peptococcaceae*	0	1
Bacillota	*Sarcina*	1	0
Bacillota	*SMB53*	1	0
Bacillota	*02d06*	1	0
Verrucomicrobia	*Akkermansia*	5	0
Pseudomonadota	*Helicobacter*	1	2
Pseudomonadota	*Desulfobacterota*	0	1
Pseudomonadota	*Sutterella*	1	0
Pseudomonadota	*Desulfovibrio*	2	1
Pseudomonadota	*Oxalobacter*	1	0
Pseudomonadota	*Sphingomonas*	1	0
Pseudomonadota	*Klebsiella*	0	1
Pseudomonadota	*Escherichia*	1	0
Pseudomonadota	*Shigella*	1	0
Actinomycetota	*Bifidobacteria*	2	0
Actinomycetota	*Enterorhabdus*	1	0
Actinomycetota	*Gardnerella*	1	0
Actinomycetota	*Johnsonella*	1	0
Actinomycetota	*Candidatus_Saccharimonas*	0	1
Fusobacteria	*Sneathia*	1	0

**Table 3:** Impact of liraglutide treatment on gut microbiota in humans.

Phylum	Genus	Increased	Decreased
Bacteroidota	*Alistipes*	0	1
Bacillota	*Lactococcus*	1	0
Bacillota	*Blautia*	0	1
Bacillota	*Dialister*	0	1
Bacillota	*Megasphaera*	0	1
Bacillota	unknown genus in the family Christensenellaceae	1	0
Verrucomicrobia	*Akkermansia*	1	0
Pseudomonadota	*Sutterella*	0	1

**Table 4:** Impact of exenatide or exendin-4 treatment on gut microbiota in animals.

Phylum	Genus	Increased	Decreased
Bacteroidota	*Odoribacter*	1	0
Bacteroidota	*Bacteroides*	0	1
Bacteroidota	*Barnesiella*	1	0
Bacillota	*Lactobacillus*	1	1
Bacillota	*Romboutsia*	0	1
Bacillota	*Streptococcus*	0	2
Bacillota	*Weissella*	0	1
Bacillota	*Marvinbryantia*	0	1
Bacillota	*Enterococcus*	0	1
Bacillota	*Lactococcus*	0	1
Bacillota	*Ruminococcus*	1	0
Bacillota	*Flavonifactor*	0	1
Verrucomicrobia	*Akkermansia*	1	0
Actinomycetota	*Enterorhabdus*	0	1

**Table 5:** Impact of exenatide treatment on gut microbiota in humans.

Phylum	Genus	Increased	Decreased
Actinomycetota	*Bifidobacterium*	1	0
Bacteroidota	*Prevotella*	1	0
Bacillota	*Oscillospira*	0	1
Bacillota	*Flavonifractor*	0	1
Bacillota	*Lactococcus*	0	1
Bacillota	*Anaerotignum*	0	1
Bacillota	*Coprococcus*	1	0

**Table 6:** Impact of dulaglutide treatment on gut microbiota in animals.

Phylum	Genus	Increased	Decreased
Bacteroidota	*Alistipes*	1	0
Bacteroidota	*Bacteroides*	1	0
Bacteroidota	*Barnesiella*	1	0
Bacteroidota	*Parabacteroides*	1	0
Bacillota	*Aerococcus*	1	0
Bacillota	*Clostridium sensu stricto*	1	0
Bacillota	*Clostridium XlVb*	1	0
Bacillota	*Coprobacillus*	0	1
Bacillota	*Enterococcus*	1	0
Bacillota	*Lachnospiracea_ incertae_sedis*	1	0
Bacillota	*Macrococcus*	0	1
Bacillota	*Oscillibacter*	1	0
Bacillota	*Pseudoflavonifractor*	1	0
Bacillota	*Roseburia*	0	1
Bacillota	*Ruminococcus*	1	0
Bacillota	*Streptococcus*	0	1
Pseudomonadota	*Escherichia/Shigella*	1	0
Pseudomonadota	*Parasutterella*	1	0
Verrucomicrobia	*Akkermansia*	1	0

**Table 7:** Impact of dulaglutide treatment on gut microbiota in humans.

Phylum	Genus	Increased	Decreased
Bacteroidota	*Bacteroides*	1	0
Bacteroidota	*Prevotella*	1	1
Bacillota	*Blautia*	0	1
Bacillota	*Lactobacillus*	1	0
Bacillota	*Ruminococcus*	0	1
Actinomycetota	*Bifidobacterium*	1	1

**Table 8:** Impact of semaglutide treatment on gut microbiota in animals.

Phylum	Genus	Increased	Decreased
Bacteroidota	*Bacteriodes*	1	0
Bacteroidota	*Odoribacter*	0	1
Bacteroidota	*Muribaculaceae*	1	0
Bacteroidota	*Alloprevotella*	1	0
Bacteroidota	*Alistpes*	1	0
Bacillota	*Blautia*	1	0
Bacillota	*Dubosiella*	2	1
Bacillota	*Romboutsia*	0	2
Bacillota	*Enterococcus*	1	0
Bacillota	*Lactobacillus*	1	1
Bacillota	*Clostridia _UCG_014*	1	0
Bacillota	*Ligilactobacillus*	0	1
Bacillota	*Allobaculum*	1	0
Bacillota	*Lachnospiraceae_NK4A136_group*	0	1
Bacillota	*unclassified_f_Oscillospiraceae*	0	1
Pseudomonadota	*Escherichia-Shigella*	1	0
Pseudomonadota	*Helicobacter*	1	0
Actinomycetota	*Coriobacteriaceae UCG-002*	2	0
Verrucomicrobia	*Akkermansia*	2	0

## Abbreviations

GLP-1—glucagon-like peptide-1, GLP-1RAs—glucagon-like peptide-1 receptor agonists, PRISMA—preferred reporting items for systematic reviews and meta-analyses, T2D—type 2 diabetes, CKD—chronic kidney disease, HFD—high fat diet, NASH—non-alcoholic steatohepatitis.
